# P-429. Infants Are Not Just Little Children: Population PK Modeling Reveals Age-Dependent Differences in Cephalexin Absorption and Clearance

**DOI:** 10.1093/ofid/ofaf695.645

**Published:** 2026-01-11

**Authors:** Andrew Haynes, Peter Anderson, Daniel Gonzalez, Kevin Messacar

**Affiliations:** University of Colorado School of Medicine, Children's Hospital Colorado, Aurora, Colorado; Skaggs School of Pharmacy and Pharmaceutical Sciences / University of Colorado Anschutz Medical Campus, Aurora, Colorado; Duke University School of Medicine, Durham, North Carolina; University of Colorado, Children’s Hospital Colorado, Aurora, Colorado

## Abstract

**Background:**

Cephalexin is a widely used oral antibiotic in pediatrics, but the pharmacokinetic (PK) processes driving drug exposure—particularly absorption and clearance—change with age. These maturational shifts may influence inter-individual variability (IIV) and pharmacodynamic (PD) target attainment. We developed a combined population PK (popPK) model to characterize how age-related changes in absorption rate (Ka), lag time (Tlag), and clearance (CL) affect cephalexin exposure from early infancy to adolescence.

**Table 1:**
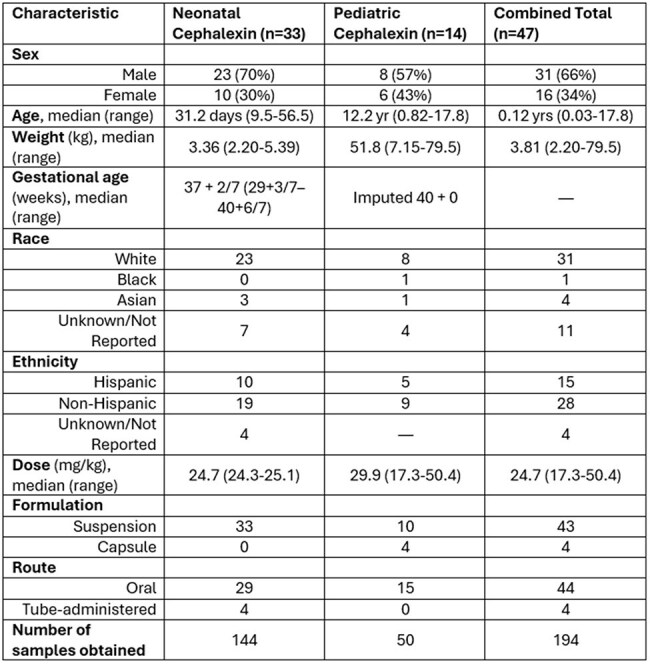
Combined Study Population Demographics

**Figure 1:**
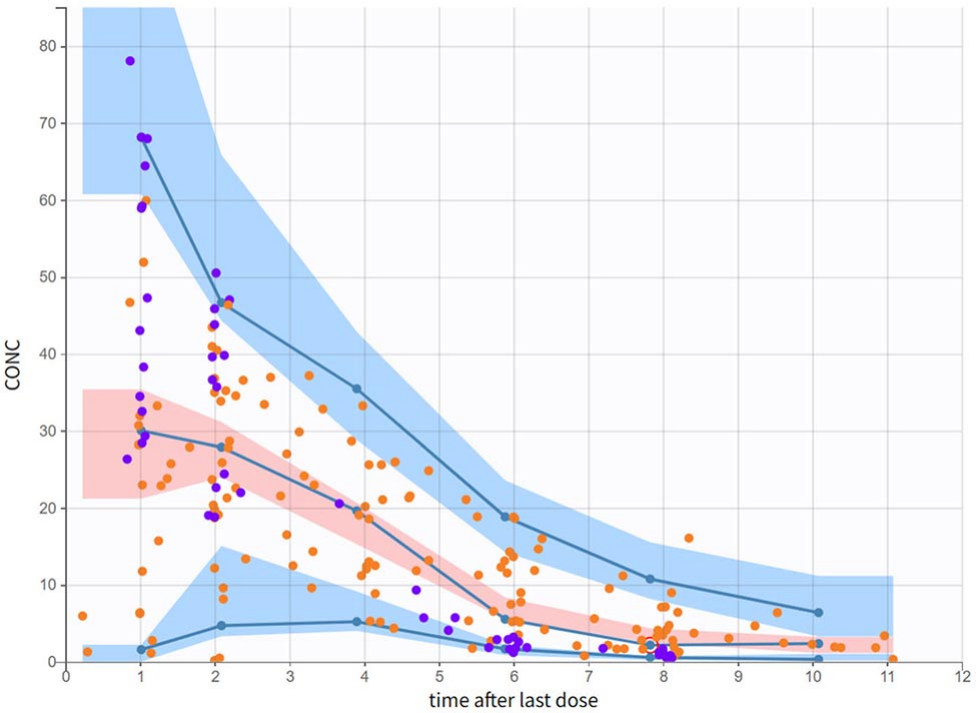
Visual Predictive Check Visual predictive check for the final PK model showing the 5th, 50th, and 95th percentiles of observed data (solid lines) and the 90% prediction intervals for the simulated data (shaded areas). Observed data are stratified by age, with the neonatal population in orange and the older pediatric cohort in purple.

**Methods:**

We combined two prospectively collected cephalexin datasets: one from neonates (0-60 days) and one from older children with osteomyelitis. A single popPK model was developed using nonlinear mixed-effects modeling. Covariate selection followed stepwise forward inclusion and backward elimination (p < 0.05 and p < 0.01, respectively). Simulations assessed the probability of target attainment (PTA) for various *f*T > MIC targets across a range of MICs, doses, and ages. Additional sensitivity analyses varied Ka and CL to explore their relative impact on PTA.

**Results:**

The final model included 194 concentrations from 47 subjects (33 infants, 14 older children). Postnatal age was a covariate on Ka. CL was modeled using weight and postmenstrual age (PMA) via allometric scaling and a sigmoidal maturation function. Absorption was slower and often delayed in neonates, while CL increased with weight and PMA. Simulations showed higher PTA in neonates at equivalent mg/kg doses, largely due to slower CL. However, absorption differences influenced PTA, especially in younger infants. For example, in full-term neonates ≤28 days, varying Ka across its simulated range altered PTA by ∼25% for a PD target of 70% fT > MIC = 8 mg/L, while varying CL changed PTA by ∼60%. Estimated IIV was higher in neonates, especially for absorption parameters, though this may reflect both biological variability and methodological factors such as sparser sampling or model uncertainty.

**Conclusion:**

This unified popPK model captures developmental changes in oral cephalexin PK across childhood. As expected, CL had the most significant effect on PTA, but slower absorption kinetics in neonates is an underrecognized contributor to PTA. These findings support age-specific, model-informed dosing of cephalexin.

**Disclosures:**

Peter Anderson, PharmD, Gilead: Grant/Research Support Daniel Gonzalez, PharmD, PhD, Melinta Therapeutics: Advisor/Consultant|Simulations Plus: Advisor/Consultant|Superluminal Medicines: Advisor/Consultant|Tellus Therapeutics: Advisor/Consultant

